# Suspended sediments limit coral sperm availability

**DOI:** 10.1038/srep18084

**Published:** 2015-12-14

**Authors:** Gerard F. Ricardo, Ross J. Jones, Peta L. Clode, Adriana Humanes, Andrew P. Negri

**Affiliations:** 1 Centre for Microscopy, Characterisation and Analysis, The University of Western Australia, Perth, Western Australia, 6009, Australia; 2 Australian Institute of Marine Science, Townsville, 4180, Queensland, and Perth, 6009, Western Australia, Australia; 3 Western Australian Marine Science Institution, Perth, 6009, Western Australia, Australia; 4 Oceans Institute, The University of Western Australia, Perth, Western Australia, 6009, Australia; 5 Australian Research Centre of Excellence for Coral Reef Studies, James Cook University Townsville, Queensland, Australia

## Abstract

Suspended sediment from dredging activities and natural resuspension events represent a risk to the reproductive processes of coral, and therefore the ongoing maintenance of reefal populations. To investigate the underlying mechanisms that could reduce the fertilisation success in turbid water, we conducted several experiments exposing gametes of the corals *Acropora tenuis* and *A. millepora* to two sediment types. Sperm limitation was identified in the presence of siliciclastic sediment (230 and ~700 mg L^−1^), with 2–37 fold more sperm required to achieve maximum fertilisation rates, when compared with sediment-free treatments. This effect was more pronounced at sub-optimum sperm concentrations. Considerable (>45%) decreases in sperm concentration at the water’s surface was recorded in the presence of siliciclastic sediment and a >20% decrease for carbonate sediment. Electron microscopy then confirmed sediment entangled sperm and we propose entrapment and sinking is the primary mechanism reducing sperm available to the egg. Longer exposure to suspended sediments and gamete aging further decreased fertilisation success when compared with a shorter exposure. Collectively, these findings demonstrate that high concentrations of suspended sediments effectively remove sperm from the water’s surface during coral spawning events, reducing the window for fertilisation with potential subsequent flow-on effects for recruitment.

Dredging to create or widen shipping channels is a largely unavoidable component of most port and coastal infrastructure developments^1^. Sediments released into the water column by dredging and natural resuspension events can migrate over nearby benthic communities where they can have pronounced effects on organisms such as coral^2^,^3^,^4^, seagrass^5^, fish and invertebrates^6^,^7^. Understanding the potential environmental effects of dredging is now particularly important in tropical Australia where a resources boom has increased demand for further development of large-scale coastal infrastructure facilities and ports^8^. For example, 200 M m^3^ of sediments along the Western Australian coastline and an estimated 85 M m^3^ of sediment in or around the Great Barrier Reef Marine Park are proposed to be dredged over the next 25 years to allow ship access to these facilities^9^,^10^.

Dredging and natural resuspension events release sediments into the water column and once disturbed, fine silts and clays can remain in suspension for extended periods and travel distances of 100s of km^11^,^12^. The effect on adult coral colonies can be significant, with sediments causing decreased productivity and growth rates^13^,^14^,^15^, and subsequent sediment smothering causing tissue damage and mortality^16^,^17^,^18^. Consequently, coral loss can occur when dredging occurs in the proximity of reefs^19^,^20^.

The early life-history stages of corals are also known to be susceptible to elevated suspended sediments (SS) (see review by Jones, *et al.*^21^), and there is a great deal of attention to these effects, as reproduction and recruitment underpins the maintenance and resilience of reef communities, and poor water quality conditions during spawning periods could lead to loss of the entire reproductive output for the year^22^,^23^,^24^. Most scleractinian corals are broadcast spawners, releasing gametes 1–2 times a year and often over a few nights in synchronous, multispecific mass spawning events^25^,^26^,^27^. Gametes are released packaged in a positively buoyant bundle that dissociate within an hour upon reaching the water’s surface, releasing sperm and eggs^28^,^29^. Mixing of gametes by wind, waves, and currents occurs at or just under the water surface promoting out-crossing but also causing sperm concentrations to rapidly dilute. Sperm dilution combined with the deleterious aging of the gametes, limits the opportunity for successful fertilisation to a comparatively short (<2 h) window following spawning^30^,^31^.

As a framework for evaluating the effects of sediment on coral reproduction, Jones, *et al.*^21^ recently developed a conceptual model of known and biologically plausible cause-effect pathways whereby suspended sediments could affect all aspects of the reproductive processes, from gametogenesis though spawning, embryogenesis, settlement and post-settlement survival. Of the 30+ proposed known and putative cause-effect pathways, many were associated with the fertilisation stage. Three main groups consisting of nine pathways were postulated in which elevated suspended sediment could prevent coral fertilisation. These were: (i) impacts on egg viability, (ii) impacts on sperm viability, and (iii) reduced egg-sperm encounters and sperm penetration into the egg. These potential mechanisms are depicted in the conceptual diagram of Fig. 1.

Several studies have reported that SS can reduce fertilisation success at concentrations as low as ≥50 mg L^−1 ^32,33,34, i.e. within an environmentally relevant range associated with dredging projects^21^. These studies included species from the genus *Acropora*, which are typical of clear water as well as *Pectinia lactuca*, which is common in turbid water habitats. However, some experiments have not detected any effects at SS concentrations as high as ~1000 mg L^−1 ^34. The reason for these marked differences in sensitivity is not known, but could be linked with sediment geochemical parameters (including effects of sediment-bound contaminants), particle grain size and nutrient content^21^,^34^. Further, differences in responses could be species-specific, or to differences in the experimental conditions under which the tests were conducted^21^. For example, one likely source of the variation between studies is the use of different sperm concentrations, which can significantly influence the outcome of fertilisation experiments^35^,^36^. Fertilisation success in corals increases non-linearly with sperm concentration because of an increase in egg-sperm encounters, resulting in maximum fertilisation success at 10^5^–10^7^ sperm mL^−1 ^31,37,38. In fertilisation assays, the effect of a treatment is usually more pronounced at lower sperm concentrations as inhibitory effects can be masked at higher, saturating sperm concentrations^36^. For corals, few toxicology studies have attempted to match the range of ecologically relevant sperm concentrations that occur *in situ*^39^,^40^. A number of approaches have been proposed to address this issue including use of a number of sperm concentrations in assays, and a calculation of two metrics which describe maximum fertilisation (*F*_max_) and the sperm concentration required to reach this maximum [Sperm]_max_^35^. Further, determination of the EC_50_ (sperm concentration required for half the maximum fertilisation, *F*_max_) has been used when data are non-linear and asymptotic^39^,^41^. Another equally important source of variability between studies could be gamete viability and quality which can vary between spawning nights and decrease quickly following release from the bundle^31^,^42^.

To improve risk assessments of the effects of dredging and turbidity-generation near reefs during spawning events, and to better understand the source of the variability between past studies, there needs to be an improved understanding of the mechanism(s) by which sediment reduces fertilisation success. In this study, we examine cause-effect pathways for the effect of sediments (relevant to dredging and natural resuspension events) on coral gametes through a series of manipulative experiments. We test the hypothesis that greater sperm concentrations would be required to attain maximum fertilisation under the presence of environmentally realistic suspended sediment concentrations and test how prolonged exposure of gametes to suspended sediment concentrations before fertilisation may amplify failure as gametes aged or were reduced in number. The principle finding, that sediments primarily impact sperm rather than eggs, is then discussed with respect to water quality conditions that can occur during dredging projects and natural turbidity events such as wind and wave resuspension from storms and cyclones.

## Results

### SS generation during dredging operations and cyclones

In order to select dredging-relevant SS concentrations for the series of experimental exposures, turbidity data from instantaneous nephelometer measurements at two sites during the Barrow Island dredging (see Methods) were assessed. SS concentrations derived from the turbidity measurements during dredging activities regularly exceeded ~100 mg L^−1^ and on occasions >300 mg L^−1^ (Fig. 2a). Similar SS concentrations were measured during a natural resuspension event (category 4 Cyclone Bianca (2011)) which passed approximately 100 km to the west of the dredging location. Dredging was temporarily suspended for a few days during the cyclone and when cyclones Dianne (2011) and Carlos (2011) were in close enough proximity to have affected safety. All dredging activities were stopped for a further 12 day period (20 March to 31 March 2011) during the predicted autumn spawning period to comply with the mandatory coral spawning impact minimization window^21^,^43^. Over these shutdown periods suspended sediment concentrations were reduced dramatically, largely to baseline (pre-dredging) levels (Fig. 2a). The maximum turbidity peaks were also similar to those recorded during cyclones and notably a category 3 Tropical Cyclone (Olwyn) which passed by the Pilbara coast of Western Australia during the first night of the coral spawning period (12–15 March 2015) causing turbidity levels to exceed 275 NTU for 24 h (pers. comm. - Travis Elsdon) (Fig. 2b–d).

### Influence of SS on fertilisation at multiple sperm concentrations

Fertilisation success increased non-linearly with sperm concentration (Fig. 3a–c). For siliciclastic (≤10 μm) sediment, fertilisation curves shifted to the right in the presence of both high and low sediment concentrations. The difference in the EC_50_ values (sperm concentration required for half the maximum fertilisation, *F*_max_) was statistically significant and ranged from ~2 fold greater at the low sediment treatment (F_1, 43_ = 5.55, P=0.023) to 37 fold greater in the high sediment treatment (high: F_1, 53_ = 363.90, P = <0.001) (Table 1). No significant difference was detected between the EC_50_ for the carbonate (≤10 μm) sediment and control curves (F_1, 43_ = 0.13, 0.721). Maximum fertilisation (*F*_max_) for all sediment treatments were high (>90% fertilisation) and there was no significant difference in comparison with the controls (siliciclastic (high): t_8_ = 1.16, P = 0.281, siliciclastic (low): t_6_ = 0.71, P = 0.507, carbonate: t_6_ = 1.19, P = 0.278), indicating that sediment did not affect egg viability. There was no difference in fertilisation success between the agitated controls on the rollers and the non-agitated controls in the 6-well plates (F_1, 41_ = 0.005, P = 0.946).

### Sperm counting in the water’s surface with SS

Flow cytometry revealed a ~10% (at 10^6^ sperm mL^−1^) to 50% (at 10^4^ and 10^5^ sperm mL^−1^) decrease in sperm numbers in the absence of sediment following 30 min of agitation, probably caused by the negative buoyancy and some clustering and flocculation of the sperm. There were further significant (22%–43%) decreases in surface sperm counts across the range of initial sperm concentrations for siliciclastic SS treatments (Fig. 4a and Table 2). A decrease of 14%–26% sperm counts was observed with carbonate sediment but this was only statistically significant at the low sperm concentration (10^4^ sperm mL^−1^).

To assess whether reductions in sperm at the surface caused by suspended sediment quantitatively accounts for observed effects on fertilisation, the expected rates of fertilisation caused by sperm dropout (Fig. 4a) were calculated by interpolation of reduced sperm concentrations into the control fertilisation curves in Fig. 3. The observed fertilisation in the presence of SS was then plotted against the fertilisation expected from sperm dropout (Fig. 4b). Once all samples were standardised to their initial sperm concentration, the reduction of sperm was generally proportional to the drop in the fertilisation. The exceptions were with carbonate sediment at initial sperm concentrations of 10^4^ and 10^5^ sperm mL^−1^, where greater fertilisation occurred than expected for the reduction in sperm observed.

When sperm were added to suspended sediments, small flocs appeared on the bottom of the chambers (Fig. 5a). Scanning electron microscopy images revealed *A. tenuis* sperm tangled, coated, buried, and damaged in aggregations of fine sediment grains (Fig. 5b). Similar inspection of the eggs revealed very few sediment grains bound to the outer surface (Fig. 5c).

### Gamete exposure duration of SS on fertilisation

There was a significant effect of siliciclastic sediment on fertilisation success of *A. millepora* at both sperm concentrations (5 × 10^4^ sperm mL^−1^: F = 9.18, P = < 0.007; 10^5^ sperm mL^−1^: F = 38.12, P = < 0.001) but the effect was more pronounced when the gametes had been exposed for longer durations (120 min), resulting in a 19% greater decrease at 5 × 10^4^ sperm mL^−1^ and a 46% greater decrease at 10^5^ sperm mL^−1^ (Fig. 6a). In the absence of SS, fertilisation success halved (51%) for gametes at the high sperm concentration but decreased by 76% at the lower sperm concentration. This decrease in fertilisation success at the low sperm concentration in the absence of sediments meant the impact of sediment was not as pronounced, because fertilisation success was already low (21%). After a long exposure to sediments, both sperm concentration triggered almost no fertilisation success (3%).

The contraction of the fertilisation window with suspended sediments and gamete aging is described schematically in Fig. 6b. Under sediment-free conditions, fertilisation peaks and gradually decreases over time as the slick dilutes and gametes age. The effects of SS are amplified under decreasing sperm concentrations and gamete viability, mostly impacting the tail of the window and causing a contraction to the left. Under extreme sediment concentrations, the maximum fertilisation rate is heavily impacted in addition to leftward contraction of the fertilisation window.

## Discussion

By applying several lines of evidence we propose sperm coagulates with sediment particles, contributing to reduced numbers of sperm at the water surface, and subsequently to sperm limitation and reduced fertilisation. These effects are more pronounced (i) at lower sperm concentrations, (ii) for siliciclastic sediments and (iii) following prolonged exposures of aging gametes to suspended sediments, and each of these factors in combination can impact fertilisation success and can contract the fertilisation window.

**Figure 1:**
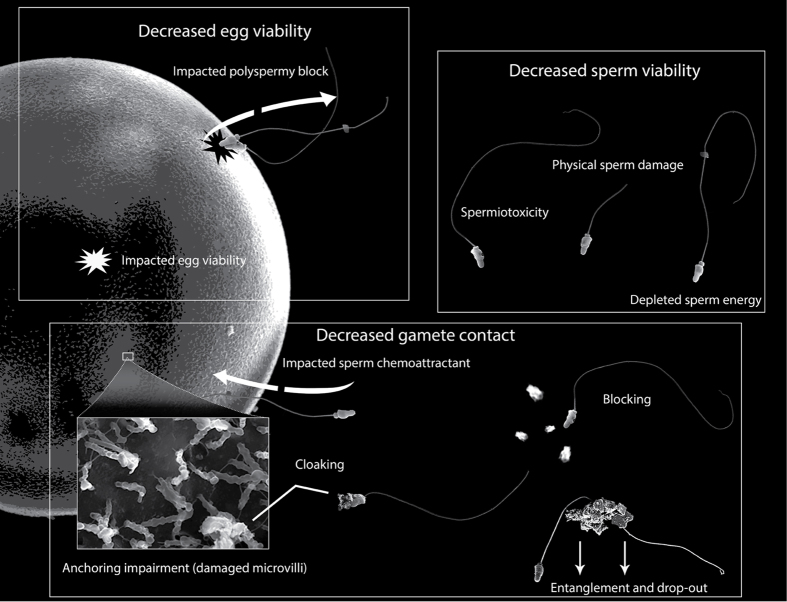
Possible cause-effect pathways in which suspended solids (SS) can reduce fertilisation rates in broadcast spawning corals. Clockwise from top left, SS could impact egg viability through damage to the egg or through impacting the polyspermy block. Similarly, SS could impact sperm viability from toxic substances leached from the sediment, physically through abrasion, or by depleting energy reserves via increased flagellum beating. SS could also decrease egg-sperm contacts and sperm penetration by damage to anchoring protrusions on the egg (microvilli), by impairing the sperm chemoattractant, by cloaking of the gametes, slowing and blocking sperm, and by entanglement and sinking of the sperm.

Elevated siliciclastic sediments had marked effects on fertilisation success at moderate sperm concentrations (<10^6^ sperm mL^−1^), increasing the EC_50_ (half the maximum fertilisation success) 2–37 fold, yet eggs were still capable of being fertilised, even at high sediment concentrations if the eggs were saturated with high sperm concentrations (10^6^ sperm mL^−1^). This suggests the mechanism affecting fertilisation success by sediment was not related to egg viability^35^. Furthermore, when examined under SEM, few sediment grains were bound or attached to the egg surface, suggesting egg cloaking was not a major cause of reduced fertilisation success (Fig. 1). Instead, the strong dependence of the fertilisation success rate on the sperm concentration suggests an occurrence of sperm limitation with sediment suspensions physically impeding the movement of the sperm or attaching to sperm and causing them to sink, therefore reducing opportunities for egg-sperm contact. The observed reduction in sperm concentration at the surface of the water in the presence of sediments is consistent with sediments vertically “stripping” sperm from upper surface (and away from the eggs). This mechanism is further supported by the microscopy showing sperm cells highly entangled with the sediment particles. The current study applied continual agitation to maintain the sediments in suspension (and mimicking water movement *in situ*), but the density of the sperm-sediment flocs was still great enough to overcome the replenishment of sperm to the surface and largely accounted for the observed reductions in fertilisation.

In addition to the physical effects of sediment on sperm there may be an additional mechanism associated with decreasing gamete viability. Eggs of the corals *Montipora digitata* and *Platygyra sinensis* remain viable for fertilisation 2 h after spawning but begin to show signs of reduced viability >2 h^31^. Here, after a short (15 min) exposure period to SS, there was a small yet significant decrease in *A. millepora* fertilisation even at high sperm concentrations; however, the effects of SS exposure on fertilisation was much more pronounced following 2 h SS exposures. However, gametes exposed for 2 h almost completely failed to fertilise, regardless of the sperm concentration. The mechanism for this further reduction in fertilisation success after prolonged exposure to SS is not known but could include damage through abrasion, impact to the polyspermy block, or a depletion of energy reserves in the sperm.

The net effect of the decrease in sperm availability and impact on aging gametes could have a significant impact on the overall fertilisation window by reducing the opportunity for successful fertilisation (Fig. 6b), similar to dilutive forces associated with wind, waves and currents. Although the impact of SS is masked at saturating sperm concentrations (>10^6^ sperm mL^−1^), these sperm concentrations *in situ* are likely fleeting because of dilutive effects and an often patchy distribution of spawning conspecifics. Only one study has provided insight into *in situ* coral sperm concentrations of a single species, *Montipora digitata*^31^. Washed eggs were fertilised with sperm samples collected from a spawning slick in relatively calm weather conditions. During the main night of spawning, high fertilisation success was recorded in a few samples indicating saturating sperm concentrations, but more often the fertilisation success was low indicating sub-optimal sperm concentrations. Further, high fertilisation rates were recorded with sperm taken after 1 h following spawning from 1 m below the water’s surface, suggesting that sperm rapidly sank. Given the rapid dilution of sperm and the aging of gametes *in situ*, the fertilisation window is limited to less than 2 h^30^,^31^. For degraded reefs with low numbers of fecund adult conspecifics, the fertilisation window is likely constrained by sperm limitation and further exacerbated by the impacts of SS.

A number of studies have stressed the importance of suitable sperm concentrations in determining effects on fertilisation in marine invertebrates, with greater sensitivity to pollutants and climate stressors reported at sub-saturating sperm concentrations^35^,^39^,^41^. The reported sensitivity of coral fertilisation to SS has varied considerably between past studies and part of this variation is likely due to differences in sperm concentration. For example, Gilmour^33^ reported significant decreases in fertilisation at just 50 mg L^−1^ SS using a low sperm concentration of 10^4^ sperm mL^−1^, whereas Humphrey, *et al.*^34^ reported no effects for some sediment types up to 1000 mg L^−1^ but using saturating sperm concentrations of 2 × 10^6^ sperm mL^−1^.

**Figure 2:**
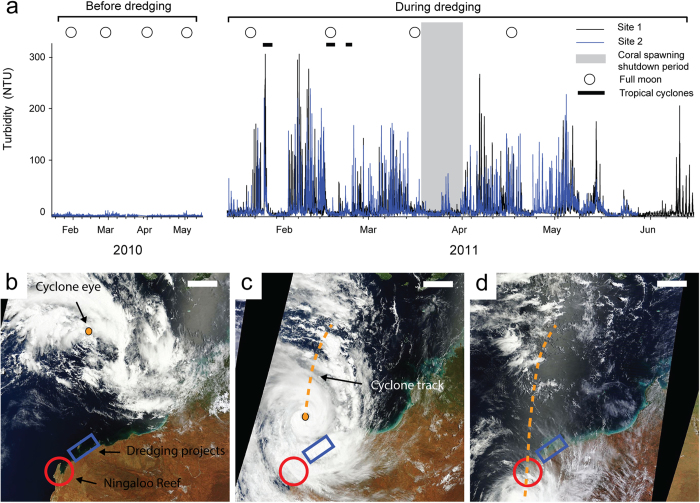
Turbidity-generating events during coral spawning periods in north-west Australia. (a) Turbidity (NTU) readings at two water quality monitoring sites ~300 m north (Site 1) and south (Site 2) during the autumn (coral spawning) months in 2010 (before dredging) and 2011 during dredging a major capital dredging program (~7.6 M m^3^ of sediment dredged over 530 d) at Barrow Island. The autumn full moons are indicated and the shaded area indicates the major predicted autumn coral spawning period 8 days after the full moon. Dredging operations were suspended for 12 days from 20–31 March 2011 for the coral spawning environmental window and for a few days associated with the close proximity of cyclones Bianca, Dianne and Carlos. (**b–d)** Moderate Resolution Imaging Spectroradiometer (MODIS) images from the Terra Satellite of Category 3 Tropical Cyclone Olwyn passing through Western Australian coastline from 10–13 March. Cyclone Olwyn developed in the Indian Ocean approximately 800 km from the Kimberley and Pilbara and hit the Ningaloo coastline on 12–13 March 2015, coinciding with the March coral spawning event. Scale bars = 200 km. We acknowledge the use of Rapid Response imagery from the Land, Atmosphere Near real-time Capability for EOS (LANCE) system operated by the NASA/GSFC/Earth Science Data and Information System (ESDIS) with funding provided by NASA/HQ.

**Figure 3:**
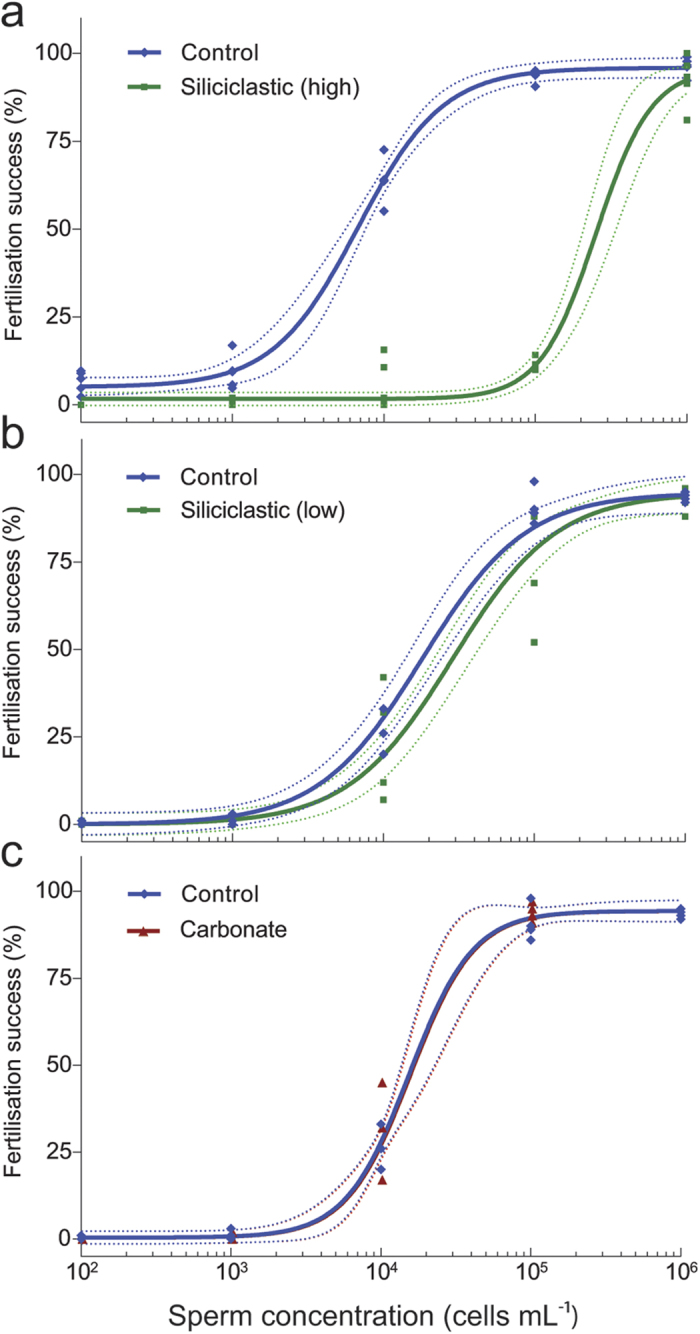
*Acropora tenuis* fertilisation success (%) curves fitted to four-parameter logistic models plotted over a range of sperm concentrations (10^2^–10^6^ sperm mL^−1^). (**a**) Siliciclastic (high): 705 mg L^−1^ , green; control: 0 mg L^−1^, blue and (**b**) siliciclastic (low): 230 mg L^−1^, green; control: 0 mg L^−1^, blue) and (**c**) carbonate: 230 mg L^−1^, red; control: 0 mg L^−1^, blue) suspended solid sediment concentrations. Dashed lines represent 95% confidence bands.

**Figure 4:**
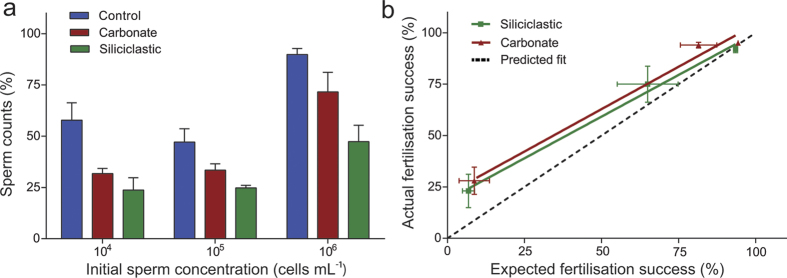
(**a**) Bar chart of *Acropora tenuis* sperm counts (means ± SE) in the water’s surface (top 1 cm) as a proportion of the initial sperm concentration after 30 minutes exposure to suspended sediments (230 mg L^−1^). *n* = 3. (**b**) Means (SE) of interpolated sperm counts (expected) with fertilisation success data (actual).

**Figure 5:**
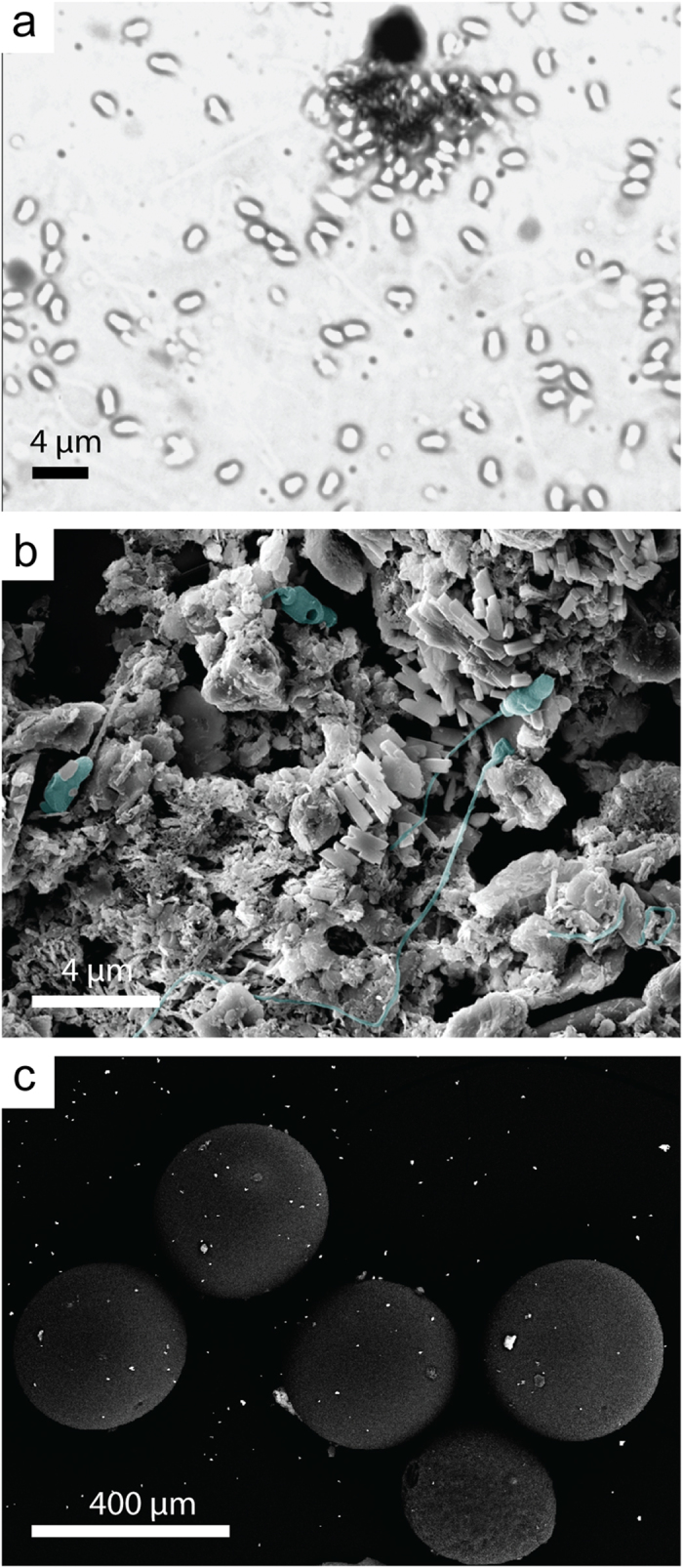
Optical and scanning electron microscopy images showing *Acropora tenuis* sperm and eggs after 30 min exposure to suspended solids. (**a)** optical microscopy image of sperm aggregating around a sediment floc, (**b)** secondary electron image of sperm (artificially coloured blue) tangled in sediment clumps, (**c**) back-scattered electron image of eggs, showing fine particles of scattered sediment, but the eggs are largely sediment free.

**Figure 6:**
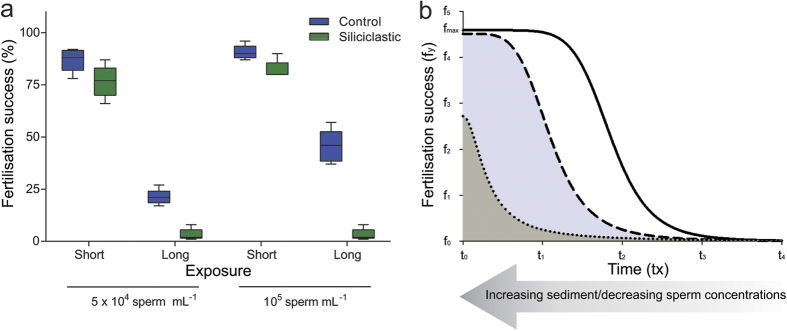
(**a**) Boxplots of *Acropora millepora* fertilisation success after short (15 min) and long exposure (120 min) periods to siliciclastic suspended solids (230 mg L^−1^) and control (0 mg L^−1^) treatments at two sperm concentrations (5 × 10^4^ sperm mL^−1^ and 10^5^ sperm mL^−1^). (**b**) A schematic diagram of changes to the fertilisation window (fertilisation success over time) with increasing sediment concentrations and decreasing sperm concentrations. The relative changes of the fertilisation window are the represented by the areas under the curves. The unbroken line (–) is typical of a normal fertilisation window, with fertilisation decreasing with gamete viability over time. As sediment concentration increases, which has the same effect as decreasing sperm concentration, the curve is moved to the left, shown by the dashed line (––). If sperm numbers are very limited, through high sediment concentrations or sperm dilution, the curve (–––) moves further left and the maximum fertilisation success (*F*_max_) decreases.

The purpose of the present study was to identify mechanisms by which sediments can affect fertilisation and we applied relatively high concentrations of SS to ensure well-defined impacts on gametes. Nevertheless, SS concentrations were guided by water quality analyses during major capital dredging projects in the Pilbara region of Western Australia in addition to other instances of SS recorded during natural and anthropogenic sediment plumes^21^,^44^,^45^,^46^. In Australia, one management strategy to reduce coral spawning slicks encountering sediment plumes is a mandatory shutdown period of dredging operations during coral spawning periods (“environmental window”) employed by regulatory authorities of Western Australia and the Great Barrier Reef Marine Park^21^,^43^,^47^. The rapid improvement in water quality associated with such a shutdown period is clearly evident in Fig. 2; however, given the costs associated with shut-down periods, it is important to consider whether partial shutdowns guided by conservative water quality guidelines might be equally protective of coral spawning success. Answering this question and effectively quantifying the risk associated with elevated sediment requires further studies determining (and spatially modelling) statistical metrics such as EC_10_ and EC_50_ for fertilisation across a range of sediment types. For example, carbonate sediments tend to have far less impact on coral fertilisation than nearshore terrigenous sediment types (this study and Humphrey, *et al.*^34^). The present study highlights the need for these future studies to carefully consider the appropriate choice of sperm concentration(s) and use a wide range of sediment concentrations, otherwise the effect of the treatment could be masked by sperm-saturation effects. Fertilisation experiments with sediments are challenging due to difficulties in simultaneously maintaining sediments and fragile gametes in suspension over the duration of experiment. Irrespective of the methods used, both the initial and final suspended sediment concentrations should be measured, as some flocculation is likely given sperm-sediment interactions. Moreover, selection of a suitable particle grain size for the experiment is fundamental and sediment leachate, contaminant and associated nutrient concentrations should be assessed as part of normal ecotoxicological procedures^48^. This approach would also benefit assessments of the potential environmental effects of wind-driven wave resuspension of terrestrially-derived sediments on the fertilisation success of corals^49^. Optimizing the coral fertilisation assays using the techniques described here should improve assessments of the environmental implications of these natural turbidity events.

In summary, sperm limitation through physical effects such as entanglement with sediment particles and stripping from the surface was identified as the key mechanism preventing and reducing successful fertilisation of coral eggs exposed to elevated suspended sediments. This finding may also have implications for other broadcast spawning marine organisms, such as fish, molluscs and echinoderms^50^. When combined with natural factors such as decreasing gamete viability and dilutive effects, the opportunity for fertilisation may be substantially reduced in the presence of sediment. As fertilisation is a critical step in recruitment, a decrease in fertilisation success may result in demographic bottleneck, reducing ongoing population maintenance or replenishment following disturbances. Furthermore, as coral spawning is ephemeral and usually confined to just a few nights, it is important that these fertilisation and recruitment processes are not inhibited.

## Materials and Methods

### Choice of suspended sediment (exposure) concentrations

SS in a shallow coral habitat during a major capital dredging program (~7.6 M m^3^ of sediment dredged over 530 d) at Barrow Island Western Australia was monitored indirectly by submerged nephelometers taking turbidity (NTU) readings every 10 minutes, positioned 1–2 m above the seabed at two water quality monitoring sites ~300 m north (site 1, 20.829 °S, 115.509 °E) and south (site 2, 20.822 °S, 115.511 °E) during the autumn (coral spawning) months in 2010 (before dredging) and 2011 (during dredging) (see Ministerial statement 800 searchable on the WA EPA website www.epa.wa.gov.au for more details) (Fig. 2). NTU recordings were made with a sideways mounted optical backscatter device (nephelometer) that can be used to estimate suspended sediment concentrations (as mg L^−1^) by applying site specific algorithms (conversion factors) based on gravimetrically determined TSS levels versus nephelometer readings of 1.3–1.6 NTU = 1 mg L^−1 ^51. Benthic sediments before dredging were predominantly unconsolidated, undisturbed carbonate with a low total organic content of <0.8% (w/w), which formed a thin veneer (0.5–3 m thick) overlying limestone pavements ranging from rubble to typically gravelly sand mixed with fine silts and clays.

### Sediment preparation and analysis

Experiments were conducted with two different types of marine sediments, a terrestrially-influenced siliciclastic (~50% quartz) sediment that we describe as “siliciclastic”, with relatively high concentrations of iron and aluminum (collected from 10 km offshore in the Pilbara region of Western Australia and in an area subject to the influence of the Ashburton River). The second type of sediment was primarily biogenic calcium carbonate (~80% aragonite), typical of offshore reefal sediment of the Great Barrier Reef (collected from Davies Reef on the Great Barrier Reef, Queensland, see Supplementary Table S1 for chemical analyses and collections locations). The sediments, hereafter referred to as siliciclastic and carbonate, were screened and milled to <63 μm then mixed with 0.4 μm filtered seawater (FSW) to create a 5 g L^−1^ suspension. To remove larger particles, the sediment was allowed to settle for 10 min and the top three-quarters of the suspension was siphoned off for use in the experiments. This reduced the modal peak of the particle size distribution to ≤ 10 μm, which is a typical grain size likely to be found at the water’s surface. To further concentrate the stock solution, the sediment was left to settle overnight and the supernatant removed. Sediment concentrations were measured gravimetrically by taking three 100 mL replicate samples using 0.4 μm polycarbonate filters and dried overnight at 60 °C. Turbidity was measured with a nephelometer (TPS 90FL-T) and a linear NTU-SS relationship was determined. During experiments, desired sediment suspensions were created by mixing the sediment stock with FSW. The final concentration of each experimental chamber was calculated by taking the average of turbidity readings before and after the experiment. The mean SS exposures for each treatment were determined using the NTU-SS relationships. Turbidity generated by the gametes (~5 NTU) was deducted from the final turbidity reading. Salinity (35.5 ppt) and pH (8.1) did not deviate throughout the experiment and dissolved oxygen remained above >95% saturation.

### Coral collection and gamete preparation

Colonies of *Acropora tenuis* (Dana, 1846) >20 cm were collected from 3–5 m depth on 21 October 2013 and 9 October 2014 from Magnetic Island (central inshore Great Barrier Reef region, 19.157 °S, 146.861 °E). Colonies of *A. tenuis* (Dana, 1846) and *Acropora millepora* (Ehrenberg, 1834) >20 cm were collected from 3–5 m depth on the 14–17 November 2013 and 3–6 November 2014 from Trunk Reef (18.329 °S, 146.846 °E) and Davies Reefs (18.832 °S, 147.633 °E), both which are located in the central, mid-shelf region of the Great Barrier Reef. The gravid colonies were then transported to the National Sea Simulator (SeaSim) at the Australian Institute of Marine Science (AIMS) and placed in flow-through tanks at 27–29 °C according to the temperature of their natal reef. Following spawning the egg-sperm bundles were gently scooped from the surface and the eggs separated from sperm using a 100 μm mesh filter and washed five times in FSW as described by Negri and Heyward 37. Eggs of each species were selected from a single colony, while sperm from multiple colonies (3–5) were pooled and the resultant stock was counted using a hemocytometer under a compound microscope. The sperm stock was then diluted to achieve a working stock concentration of 1 × 10^8^ sperm mL^−1^ from which further dilutions were made.

### Influence of SS on fertilisation at multiple sperm concentrations

Sperm concentration-response experiments were conducted using siliciclastic SS at ~230 and 700 mg L^−1^, carbonate SS at 230 mg L^−1^ and FSW (control). For the fertilisation assays sediment concentrations of 230 mg L^−1^ and 700 mg L^−1^ were used together with a FSW (control). For each of six sperm concentrations (10^1^–10^6^ sperm cells mL^−1^) of *A. tenuis*, 48 mL of the SS treatment was transferred into ten replicate 180 mL clear polystyrene chambers. Eggs were added to half the chambers (1 mL of ~200 eggs) and the desired sperm concentration (1 mL) was added to the other half. Gametes were independently exposed to the suspended sediment treatments for 30 min, simulating the time taken for the eggs to become viable and for compatible gametes to encounter each other *in situ*^52^. Sperm were then mixed with their corresponding eggs to initiate fertilisation and placed on mechanical rollers at 0.3 revolutions s^−1^ (see Supplementary Fig. S2) located at a constant temperature room set to the temperature of their parent’s natal reef (27–29 °C). The rollers consisted of a series of 3 cm diameter cylinders rotated by an electrical motor and use of the rollers maintained a constant suspension of sediment throughout the fertilisation period. The chambers were removed from the rollers when the majority of the embryos in the controls had developed to the 4-cell stage, which for *A. tenuis*, was typically 150 min after mixing sperm with eggs. Turbidity was immediately measured using a nephelometer and then embryos and eggs were fixed using Z-fix fixative (Anatech Limited). Eggs and embryos were transferred to counting trays and the first 100 embryos or eggs in each experimental chamber were assessed for fertilisation success. To control for the possible effects of mechanical agitation, similar fertilisation experiments were conducted under stationary conditions in 6-well cell culture plates (Nunclon, Thermo Scientific) on three separate occasions. In these experiments, 1 mL of the desired sperm concentration was added to 1 mL of ~200 eggs and 8 mL of FSW. Final sperm concentrations were over the range 10^1^–10^6^ sperm mL^−1^.

### Sperm counting in the water’s surface with SS

Changes in sperm concentration of *A. tenuis* were measured using a C6 Flow Cytometer and Cflow Sampler software (Accuri Cytometers Inc.). One mL of either 5 × 10^4^, 5 × 10^5^ and 5 × 10^6^ sperm mL^−1^ were added to 50 mL of 230 mg L^−1^ siliciclastic or carbonate sediment, or FSW (control) making a sperm concentration of 10^4^–10^6^ sperm mL^−1^. Sperm-only and sediment-only controls at these concentrations were also prepared. All chambers were mechanically agitated for 30 min. From each chamber, a 1 mL subsample was taken from ~1 cm below the surface for flow cytometry. Using forward and side scatter, the regions for gating were determined by running the controls separately. Values from forward scattering are proportional to particle size and the threshold was set at 20,000 signal intensity units using a calibration of 1 μm florescent beads; side scattering is proportional to their complexity and was set at the minimum signal intensity threshold of 10 units. Two clear cluster distributions were observed for each of the sediment particles and the sperm in the sample. Three replicates of each treatment or control were measured. The flow cytometer was run to count at least 100,000 particles or 2 min.

### Microscopic examination

Subsamples from the bottom and middle of sediment-sperm samples were fixed in 1.25% glutaraldehyde and 0.5% paraformaldehyde in FSW and stored at 4 °C. Samples were dehydrated in a microwave using a graded ethanol series (70%, 90% × 2, 100%, 100% (anhydrous)) for 40 s at 250 W and then critical point dried (Polaron KE3000, Quorum Technologies) in liquid CO_2_. The dried samples were then mounted on carbon tape on aluminium stubs, coated with 3 nm platinum, and imaged using a field emission SEM (Zeiss 55-VP).

### Gamete exposure duration of SS on fertilisation

To determine if a decline in gamete viability with age compounded the effect of sediment on fertilisation success, gametes (90 min age) were exposed to a suspension (230 mg L^−1^) at two sperm concentrations for two time periods (15 and 120 min). A pilot study revealed low rates of fertilisation at sperm concentrations of 10^4^ sperm mL^−1^ for *A. millepora*; therefore, 5 × 10^4^ sperm mL^−1^ was used in addition to 10^5^ sperm mL^−1^ for the experiment. Similar to the first experiment, 1 mL of ~200 eggs and 1 mL sperm was added to 10 replicates chambers each containing 48 mL of the SS treatment or control (FSW) and gently agitated on mechanical rollers. After 15 min, half the gamete exposure chambers were mixed with their counterpart to initiate fertilisation and placed back on the mechanical rollers. The remainder were mixed at 120 min and placed on the rollers. The chambers were removed from the rollers when the majority of the embryos in the controls reached the 4-cell stage, which for *A. millepora*, was generally 180 min after mixing sperm with eggs. Turbidity measurement and fixation of the embryos and eggs were conducted as described previously.

### Design and statistical analysis

We used a similar assay technique proposed by Marshall^35^ to calculate the fertilisation maximum (*F*_max_) over a range of sperm concentrations because the effect of polyspermy in acroporid corals appears to be minor (if at all)^37^,^39^. We also calculated a second metric, the EC_50_ - the concentration that yields the half maximum fertilisation response.

Fertilisation success rates were fitted to nonlinear regression curves (four-parameter logistic models) using the program GraphPad Prism (v5, San Diego, USA). The model was constrained between 0 and 100%. All curves were tested for normality of the residuals and a replicate test was applied to assess goodness of fit. For the treatment data sets, the model and the inflection point i.e. EC_50_ values were determined with greater confidence by global fitting the parameters top, slope and bottom of the curves if there was no significant difference with the controls (p>0.05)^53^. The fertilisation maximum (*F*_max_) was compared between the sediment treatments and the controls with an unpaired t-test. Agitated assays were compared with non-agitated assays to test for any effect of the mechanical rollers on coral fertilisation.

To assess changes in sperm numbers at the water surface in the presence of sediments, the estimated number of sediment particles incorrectly occurring in the gate was deducted from the total sperm count. Changes in sperm numbers between sediment treatments and the controls were analysed with unpaired t-test using GraphPad Prism (v5, San Diego, USA). Sperm counts were compared with fertilisation success curves by first standardising the data to the initial sperm concentration and then interpolating the values onto the fertilisation success curve. To test gamete exposure durations on fertilisation success, each sperm concentration was analysed separately and fit to Generalized Linear Models (GLM) using packages lattice in R (R Development Core Team, 2014). Data exploration was done by methods described in Zuur, *et al.*^54^, with the presence of outliers investigated using Cleveland dotplots. Quassi-binomial errors and the log link function were used because the data were over-dispersed.

## Additional Information

**How to cite this article**: Ricardo, G. F. *et al.* Suspended sediments limit coral sperm availability. *Sci. Rep.* **5**, 18084; doi: 10.1038/srep18084 (2015).

## Supplementary Material

Supplementary Information

## Figures and Tables

**Table 1:** Results of data fitted to a four-parameter logistic function (Fig. 2). The curves were constrained between 0 and 100%.

	Control (0 mg L^−1^)	Siliciclastic (high) (~700 mg L^−1^)
Best-fit parameters (95% CI)		
EC_50_	6.76 × 10^3^ (5.74 × 10^3^–7.96 × 10^3^)	2.51 × 10^5^ (1.92 × 10^5^–3.28 × 10^5^)
*Top/*F*_max_ (%)	95.88 (93.03–98.72)	95.88 (93.03–98.72)
Bottom (%)	5.06 (2.38–7.74)	1.65 (0.00–3.51)
Slope	1.547 (1.09–2.00)	2.36 (1.74–2.99)
	**Control (0 mg mL**^**−1**^)	**Siliciclastic (low) (~230 mg L**^**−1**^ )
Best-fit parameters (95% CI)		
EC_50_	1.81 × 10^4^ (1.31 × 10^4^–2.50 × 10^4^)	2.87 × 10^4^ (2.05 × 10^4^–4.06 × 10^4^)
*Top/*F*_max_ (%)	92.72 (88.89–100.00)	92.72 (88.89–100.00)
*Bottom (%)	~0.00 (0.00–3.22)	~0.00 (0.00–3.22)
*Slope	1.26 (0.95–1.58)	1.26 (0.95–1.58)
	**Control (0 mg L**^**−1**^ )	**Carbonate (~230 mg L**^**−1**^)
Best-fit parameters (95% CI)		
EC_50_	1.54 × 10^4^ (1.23 × 10^4^–1.93 × 10^4^)	1.54 × 10^4^ (1.23 × 10^4^–1.93 × 10^4^)
*Top/*F*_max_ (%)	94.33 (91.19–97.48)	94.33 (91.19–97.48)
*Bottom (%)	0.54 (0.00–3.02)	0.54 (0.00–3.02)
*Slope	2.04 (1.09–3.00)	2.04 (1.09–3.00)

^*^Indicates sharing of parameters (bottom, top/*F*_max_, and slope).

**Table 2:** Difference (%) in sperm counts at the water’s surface compared with the control.

Initial sperm concentration (sperm mL^−1^)	Sediment	Difference from control (%)	t-ratio (*n *= 3)	P value
10^4^	Carbonate	26	2.938	0.0425
10^4^	Siliciclastic	34	3.279	0.0305
10^5^	Carbonate	14	1.913	0.1283
10^5^	Siliciclastic	22	3.396	0.0273
10^6^	Carbonate	18	1.822	0.1425
10^6^	Siliciclastic	43	5.008	0.0074
