# Supplementary material for: Suspended sediments limit coral sperm availability
Source: Sci Rep. 2015 Dec 14;5:18084. doi: 10.1038/srep18084 (PMC4677285; doi:10.1038/srep18084)
Supplement: Supplementary Information [file srep18084-s1.pdf]

Supplementary Information

Suspended sediments limit coral sperm availability

Gerard F. Ricardo, Ross J. Jones, Peta L. Clode, Adriana Humanes, Andrew P. Negri

Table S1. Grain and properties of sediments.

Sediment type:	Siliciclastic		Carbonate	
Collection location	21.642°S, 114.924°E		18.832°S, 147.633°E	
Electron micrograph of bulk surficial sediment (screened to <500 (µm) :	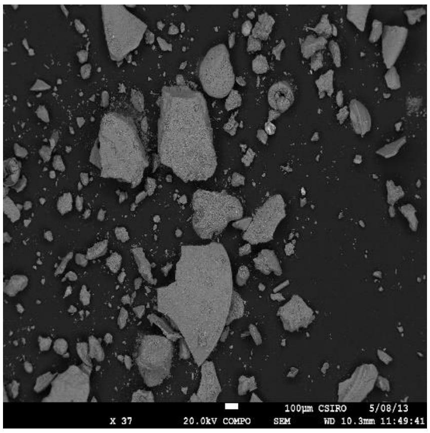		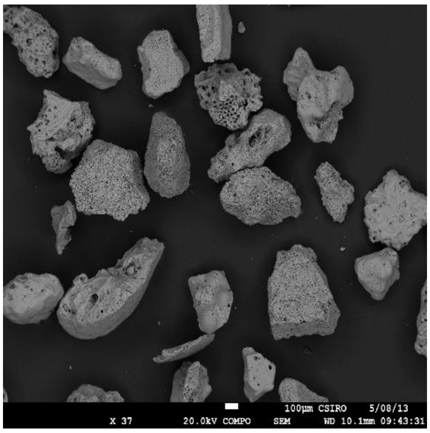	
Grain size[1] (µm)	10.41		10.36	
Median, mode (µm)	6.78, 6.32		7.69, 10.10	
Wentworth classification	fine silt (4–7.8 µm)		Fine to medium silt (7.8–15.6)	
TOC, TN (%)	0.26, 0.03		0.27, 0.03	
Geochemical parameters	Bulk sediments (%)	Dissolved metals (mg L^{-1})	Bulk sediments (%)	Dissolved metals (mg L^{-1})
Al	3.0 ± 1.6		0.143 ± 0.179	
As	<0.004		0.004 ± 0.001	
Cd		<0.002		<0.002
Cr	0.023 ± 0.028	<0.015	<0.005	<0.015
Cu	<0.002	<0.003	<0.006	<0.003
Fe	4.5 ± 1.0		0.124 ± 0.143	
Mn	0.045 ± 0.009	0.004	<0.003	0.001
Ni	0.013 ± 0.015	<0.005	<0.003	<0.005
Pb	<0.007	<0.015	<0.003	<0.015
Se		<0.05		<0.05
Ti	0.287 ± 0.078			<0.010
V	0.008 ± 0.002		<0.003	
Zn	0.004 ± 0.003	<0.002	<0.005	<0.002
